# Bioinformatics-based analysis of nicotinamide adenine dinucleotide metabolism-related genes to predict immune status and prognosis for head and neck squamous cell carcinoma patients

**DOI:** 10.3389/fimmu.2025.1609175

**Published:** 2025-06-30

**Authors:** Zhenjie Guan, Xinyu Gu, Lian Zheng

**Affiliations:** ^1^ Department of Stomatology, The First Affiliated Hospital of Zhengzhou University, Zhengzhou, China; ^2^ Department of Oncology, The First Affiliated Hospital, College of Clinical Medicine, Henan University of Science and Technology, Zhengzhou, China; ^3^ Department of Oral and Maxillofacial Surgery, The First Affiliated Hospital of Zhengzhou University, Zhengzhou, China

**Keywords:** WGCNA analysis, prognostic modeling, head and neck squamous cell carcinoma, gene mutation, immune infiltration

## Abstract

**Background:**

Patients suffering from head and neck squamous cell carcinoma (HNSCC) have a high recurrence rate and poor prognosis. Nicotinamide adenine dinucleotide (NAD^+^) is crucial in the progression of the tumor. Currently, the specific role of NAD^+^ in HNSCC remains elusive.

**Methods:**

First, weighted gene co-expression network analysis (WGCNA) was utilized to screen gene modules linked to NAD^+^ metabolism-related genes (NMRGs), and the expression profiles obtained were taken as intersections with differentially expressed genes (DEGs) between HNSCC and control samples. The genes were further compressed and risk modeled using LASSO and stepwise regression analyses. Then the gene mutation landscapes of different risk subgroups of HNSCC were analyzed using MuTect 2 software. Differences in biological function and immune infiltration analyses between different subgroups were explored. In addition, scratch and transwell assays were carried out to explore the role of *PSME1* in HNSCC cells.

**Results:**

Here, we screened two specific modules with the strongest relation to HNSCC by WGCNA and subsequently took the intersection of 6160 DEGs with the module genes, obtaining a total of 359 intersected genes. 6 (*ICOS*, *PSME1*, *SERPINA1*, *SH3KBP1*, *SP100* and *ZAP70*) characterized genes linked to HNSCC prognosis were selected for risk modeling. We categorized patients by the risk scores into high- and low-risk groups. Overall survival (OS) of patients in the low-risk group was significantly better than those in the high-risk group. Compared to the low-risk group, the mutation rates of *FAT1, TP53*, *TTN* genes were higher in the high-risk group, with a coexistence between the mutated genes. The expression of the characterized genes showed a positive association with the level immune cell infiltration, for example, activated CD8 T cells. The enrichment analysis demonstrated that differential genes in the high-risk HNSCC group were significantly enriched in the ribosome and other pathways, while the differential genes in the low-risk group were mainly involved in arachidonic acid metabolism and other pathways. Further *in vitro* assay revealed that downregulated *PSME1* attenuated the migratory and invasive abilities of FaDu cells.

**Conclusions:**

The current work provided theoretical references for future study on potential biomarkers of prognosis and immune infiltration in patients suffering from HNSCC.

## Introduction

Head and neck squamous cell carcinoma (HNSCC) ranks as the sixth most frequent malignancy globally, affecting the larynx, pharynx, and oral cavity (1, 2). It has an annual incidence of approximately 600,000 and causes over 300,000 deaths (3). Most newly diagnosed HNSCC patients are in locally advanced stages, and most of them also have regional lymph node metastasis at presentation (4–6). Although current therapies (surgical and adjuvant) have shown good progress, the 5-year survival for HNSCC patients is only around 50% because of high rates of lymphatic metastasis and postoperative recurrence (7). Human tumor virus, alcohol, and tobacco have all been identified as significant risk factors for HNSCC (8). New treatment targets have been shown to potentially enhance the results of HNSCC (9). Although some progress has been made on some of the molecular mechanisms of HNSCC, the overall pathogenesis is not yet fully understood and still needs to be further explored.

Metabolic reprogramming to promote high rates of proliferation and biomass production—both essential for tumor formation and survival—is one of the characteristics of cancer (10, 11). Cancer cells depend on glycolysis and several pathways related to glycolysis, such as serine and fatty acid synthesis, pentose phosphate pathway (PPP), glutamine catabolism, to produce macromolecules and mitigate oxidative stress caused by accelerated proliferation (12). The essential metabolite nicotinamide adenine dinucleotide (NAD^+^), which has been linked to several redox and non-redox processes, including inflammatory responses, post-translational modifications, cell signaling, senescence, apoptosis, DNA repair, is necessary for all of these up-regulated pathways (13). In addition to being a crucial coenzyme in oxidative processes, NAD^+^ is also essential for immunological response, genomic stability, cell homeostasis, and cell division and death (14, 15). During glycolysis, the cytoplasmic lactate dehydrogenase (LDH) process can produce NAD^+^, which promotes the proliferation of tumor cells. In comparison to non-cancerous cells, tumor cells have greater ratios of NADP^+^/NADPH and NAD^+^/NAD, indicating that NAD^+^ is crucial to this metabolic change (16). In addition, disturbed NAD^+^ metabolism not only affects the redox homeostasis of tumor cells, but is also closely associated with malignant phenotypes such as immune escape, therapeutic resistance, and cell proliferation in cancer (17). However, the mechanism of this role in HNSCC remains unclear.

The present study created NAD^+^ metabolism-related genes (NMRGs) model to improve the prognostic outcomes and immune infiltration of HNSCC patients. Based on the expressions of NMRGs, a prognosis model of HNSCC patients was established to separate patients into low- and high-risk groups. We also analyzed the correlation between characterized genes independently linked to HNSCC prognosis and immune infiltration based on NMRGs, and explored the differences between related signaling pathways and biological functions among different subgroups. Overall, the present work offered a new method for evaluating patient prognosis and immune infiltration based on prognosis-related features by combining several common bioinformatics algorithms for HNSCC, which offers a novel direction for the treatment and prognostic assessment for patients with HNSCC.

## Methods

### Data collection

The Cancer Genome Atlas (TCGA) database (https://portal.gdc.cancer.gov/) included the gene expression profiling, somatic mutation, and clinical phenotype data of HNSCC. The RNA-seq data were then log2 transformed and converted to TPM format. All patients were assured to have a survival time longer than 0 days, and samples with missing survival time and survival status were eliminated when processing the TCGA-HNSCC data. Screening produced 499 HNSCC samples and 44 control samples. Furthermore, the GSE41613 data was collected from Gene Expression Omnibus (GEO, https://www.ncbi.nlm.nih.gov/geo/) database. A sum of 97 tumor samples were acquired by selecting the GEO cohort with survival time, converting the probes to Symbol according to the annotation file, and excluding samples lacking clinical follow-up information and overall generation rate data. The set of NAD^+^-related genes was then acquired from the MSigDB database (https://www.gsea-msigdb.org/gsea/msigdb/index.jsp), which contained the reactome database (R-HSA-196807) and KEGG pathway database (pathway: hsa00760).

### Weighted gene co-expression network construction

Using ssGSEA in the “GSVA” package, we determined the NMRGs correlation scores for every TCGA-HNSCC sample (18). We next used the “WGCNA” package to create weighted gene co-expression network to find co-expression networks and select genes from various clusters (19, 20). All samples and missing genes are first clustered. Second, the optimal soft threshold power (β=16) is found using the “pickSoftThreshold” R function to identify significant correlations between modules more effectively. Then, using the requirement of at least 60 genes per module (minModuleSize = 60) to identify gene modules, we conducted a hierarchical cluster analysis. Last but not least, we employed the R package "Heatmap" (21) to extract various module signature genes according to the first principal component of module expression. Then, we assessed the relationship between the module genes and the diagnosis of clinical signature to test the association between module and signature scores. The genes contained in the modules were extracted by filtering the modules with the highest correlations (22).

### Identification of DEGs and enrichment analysis

In the TCGA cohort, the “limma” package was utilized to find DEGs between HNSCC and control samples (23). Using *p*-adj < 0.05 and |log 2(FC)|>log2(1.5), the gene expression profile was professionally summarized, quartile normalized, and background adjusted in order to screen for significant DEGs. Following their intersection with the DEGs, the midnightblue and green modular genes were found and examined using the R package “clusterProfiler” (24) to examine the module genes’ KEGG function and gene ontology (GO) (the screening criteria was *p*-value < 0.05). To assess the modular gene enrichment pathways and biological processes, respectively, we created bubble diagrams by charting the top 10 functions enriched to the three terms of the GO enrichment results and the top 10 enriched KEGG pathway results. We used the R package “clusterProfiler” to compute the GSEA of the high- and low-risk groups of TCGA-HNSCC to look into the pathways of various biological processes in various subgroups. The KEGG database was used the reference for enriched pathways during analysis (25).

### Risk modeling and validation

To find genes significantly linked with prognosis in TCGA-HNSCC patients (*p*<0.05), the R package “survival” (26) was used to conduct univariate Cox proportional risk regression on intersecting genes. To enhance the model generalization, 10-fold cross-validation was employed and LASSO Cox regression analysis of the R package "glmnet" (27) to compress the genes in order to maximize gene number in the risk model. Furthermore, multifactorial stepwise regression analysis was employed to check for important genes and correlation coefficients that were independently linked to the prognosis of HNSCC, and risk scores were computed for every patient. The following is the formula: Riskscore=Σβi×Expi, where Expi is the expression of each gene gathered, i is the gene expression level, and β is the associated gene’s Cox regression coefficient. Following zscore normalization, the Riskscore was used to assign the TCGA-HNSCC patients into high- and low-risk groups by the Riskscore’s optimal critical value. The R package “survminer” (28) was then utilized to conduct survival analysis between the low- and high-risk groups. Kaplan-Meier (KM) survival curves were then displayed for prognosis analysis, followed by using log-rank test to evaluate significant differences. Further, we examined the prediction of the model by displaying time-dependent receiver operating characteristic (ROC) curves using the R package “timeROC” (29) and calculated 1-, 2-, 3-, 4- and 5-year area under the curve (AUC). Finally, we validated the GSE41613 dataset using the same methodology to better validate the stability and reliability of our constructed clinical prognostic model based on risk-related gene signatures.

### Analysis of gene mutations

Since genomic mutations are closely associated with disease onset and progression (30), we analyzed each sample in the TCGA-HNSCC cohort for gene mutations. The mutation dataset of HNSCC samples was processed using MuTect 2 software (31), and the mutation profiles of the top 10 mutated genes in the low- and high-risk groups were plotted separately.

### Immunological characterization of HNSCC

The association between Riskscore and immune function in HNSCC was evaluated by analyzing the immune infiltration of the TCGA dataset samples using the R package “estimate” (32) and expressed as their respective scores (StromalScore, ImmuneScoreh and ESTIMATEScore). The association between the Riskscore of the TCGA dataset and the 10 immune cell scores was calculated using the “MCPcounter” package (33). According to the transcriptomic expression profiles of the samples, we computed the scores of 28 tumor-infiltrating immune cells (34) in the TCGA-HNSCC cohort with the ssGSEA function of the R package "GSVA".

### Cell culture and siRNA transfection

MEM medium (Gibco, USA) and DMEM medium (Gibco, Grand Island, NY, USA) were used to culture human pharyngeal squamous carcinoma cell line (FaDu) and human normal squamous epithelial cell line (NOK) ordered from Procell Life Science and Technology Co. Ltd (Wuhan, China), respectively. All cultures were added with 1% penicillin-streptomycin (Solarbio, Beijing, China) and 10% fetal bovine serum (Clark, Richmond, VA, USA). All the cells were cultured in an incubator with 5% CO_2_ at 37°C. Utilizing Lipofectamine 3000 Transfection Reagent (Thermo Fisher Scientific, Waltham, MA, USA), si-*PSME1* and negative control si-RNA were transiently transfected. si-*PSME1* sequences were as follows, sense. UGGAUUUGUACCAUUCUUCUG, antisense: GAAGAAUGGUACAAAUCCAAG.

### RNA extraction and quantitative real-time PCR

Total RNA from NOK and FaDu cells was separated applying RNA Extraction Kit (TRIzol, Invitrogen, Carlsbad, CA, USA) according to the manufacturer’s protocols. The purity and concentration of the total RNA were assessed, and cDNA templates were generated using the HiScript II kit (Vazyme, Nanjing, China). Quantitative real-time PCR (qRT-PCR) was conducted using specific primers and the KAPA SYBR^®^ FAST kit (Sigma Aldrich, St Louis, MO, USA). GAPDH was an internal control, and the 2^-ΔΔCT^ method was used for data analysis (35). **Table 1** shows the primer sequences of the specific genes.

**Table 1:** The sequences of primers for RT−qPCR used in this study.

Gene name	Forward primer	Reverse primer
ICOS	5’ CCCATAGGATGTGCAGCCTTTG 3’	5’ GGCTGTGTTCACTGCTCTCATG 3’
PSME1	5’ TGATGACCAGCCTCCACACCAA 3’	5’ TACTCTGCCTCATCCAGCTCGT 3’
SERPINA1	5’ TCTGAAGAGCGTCCTGGGTCAA 3’	5’ GATGGTCAGCACAGCCTTATGC 3’
SH3KBP1	5’ GCAGTTCGCTATCTGGCATCCT 3’	5’ GTCTGCTTGTGGTCGGATGACT 3’
SP100	5’ GGAGAAGAGCTTCAGGAAACCTG 3’	5’ GGCTTCTTGGCACACCTTTTGG 3’
ZAP70	5’ CACTACGCCAAGATCAGCGACT 3’	5’ GGCTGGAGAACTTGCGGAAGTT 3’
GAPDH	5’ TTGCCCTCAACGACCACTTT 3’	5’ TCCTCTTGTGCTCTTGCTGG 3’

### Wound-healing experiment

Scratch and transwell assays were subsequently carried out to examine the effect of *PSME1* expression on FaDu cell migration and invasion. For migration assays, collective cell migration was detected in a wound healing assay. Transfected cells were inoculated into 6-well plates (5 × 10^5^/ml). 2 ml of cell suspension was inoculated into 6-well plates and incubated with 5% CO_2_ in an incubator at 37°C. When the cell density was approximately 80%, the monolayer was scraped with a 10 μL plastic pipette tip to create a uniform wound. PBS was used to wash the monolayers, which were then incubated in a non-FBS medium. The wound edge distances between two edges of migrating cell sheet were imaged at 0 h and 48 h, respectively. All the experiments were conducted three times.

### Transwell experiments

For the invasion assay, 1 × 10^5^ cells were inoculated into the upper chamber covered with 10% Matrigel (Corning, Inc., Corning, NY, USA) for 24-h incubation. After the incubation, cells in the upper chamber were eliminated by swabbing, while those on the lower chamber were then fixed by 4% paraformaldehyde and dyed by 0.1% crystal violet solution. These migrated or invaded cells in the lower chamber were counted under a microscope using 6 different fields of view (36).

### Statistical tests

All statistical analyses were performed using Prism 8 (GraphPad Software, San Diego, CA, USA) and R software version 3.6.0 ((R Foundation, Vienna, Austria)). Wilcoxon rank-sum test was utilized to calculate the difference between the two groups of continuous variables. Correlations were calculated using the spearman method, and the log-rank test was employed to compare the survival between patients in each subgroup. *p*<0.05 was defined to be statistically different.

## Result

### WGCNA identifies gene modules associated with NMRG

**Figure 1:**
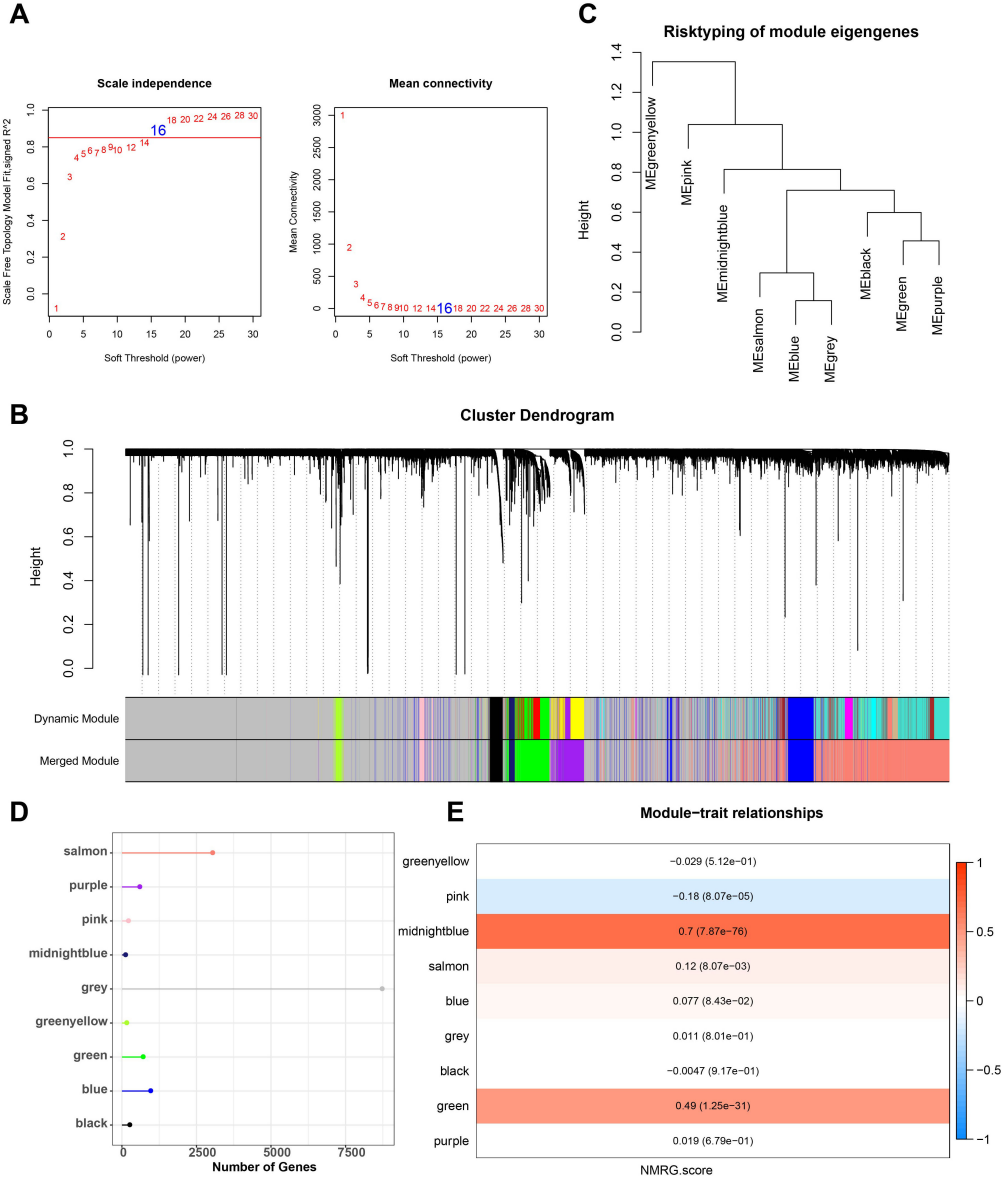
Construction of co-expression network for TCGA cohort. **(A)** Scale-free fit index analysis on different soft threshold powers (β), and average connectivity analysis on different soft threshold powers. **(B, C)** Gene dendrograms based on dissimilarity metric (1-TOM) clustering. **(D)** Gene numbers in each module. **(E)** Correlation of module eigenvectors with features for each module.

Next, we used the ssGSEA method to determine each sample’s NMRG score in the TCGA dataset. NMRG-related gene modules were identified using the R package “WGCNA”. To satisfy the scale-free topology of the network, we selected a soft threshold power of 16 to construct the topological network (**Figure 1A**). 9 co-expression modules were ultimately produced when the module correlation was computed and the module contained a minimum of 60 genes (**Figures 1B, C**). Out of the 9 modules, the grey module had a comparatively large number of genes, followed by the salmon module, as seen in **Figure 1D**. The gene that was unable to aggregate to other modules was known as the grey module. To select clinically important modules, we calculated the correlation of each module with NMRG scores and plotted a heat map of module-shape correlation. Among the nine modules, significant strong positive correlations were found between midnightblue and green modules and NMRG scores (midnightblue: cor = 0.7, *p* = 7.87e-76; green: cor = 0.49, *p* = 1.25e-31 and **Figure 1E**).

### Differential gene analysis and enrichment analysis

**Figure 2:**
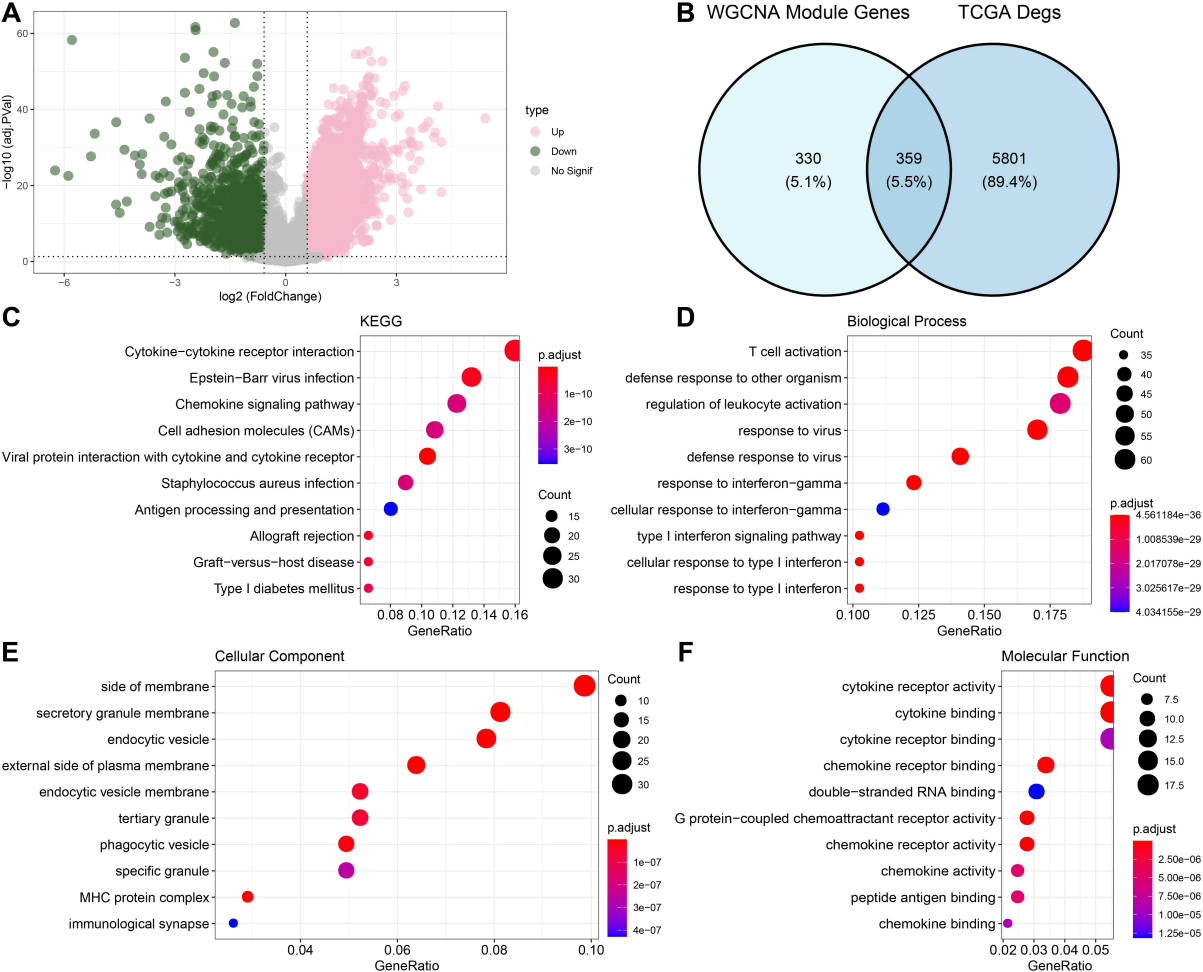
Functional enrichment analysis of DEGs with modular gene intersection genes. **(A)** Volcano map displaying the DEGs of tumor and normal group based on TCGA cohort. **(B)** The intersection of differential genes and midnightblue and green module genes. **(C-F)** Results of enrichment analysis of intersecting gene pathways.

**Figure 2A** shows the volcano map of DEGs. Next, we found DEGs between HNSCC cases and control samples in the TCGA dataset. In total, we found 1206 strongly down-regulated genes and 4954 significantly up-regulated genes. After that, we found 359 intersecting genes by taking the genes of the midnightblue and green modules and DEGs (**Figure 2B**). We used GO and KEGG to enrich the intersecting genes in order to investigate their regulatory involvement in the pathophysiology of HNSCC. According to KEGG analysis, pathways such as epstein-barr virus infection and cytokine-cytokine receptor interaction were significantly enriched with the intersecting genes (**Figure 2C**). GO enrichment analysis showed that the BPs in which the intersecting genes were largely involved were defense pathways, for instance, T cell activation and defense response to another organism (**Figure 2D**). The CCs that were mainly localized were side of the membrane, secretory granule membrane, endocytic vesicle and other structures (**Figure 2E**). The MFs most significantly enriched for intersecting genes were pathways such as cytokine receptor activity and chemokine activity (**Figure 2F**).

### Prognostic model building and validation

**Figure 3:**
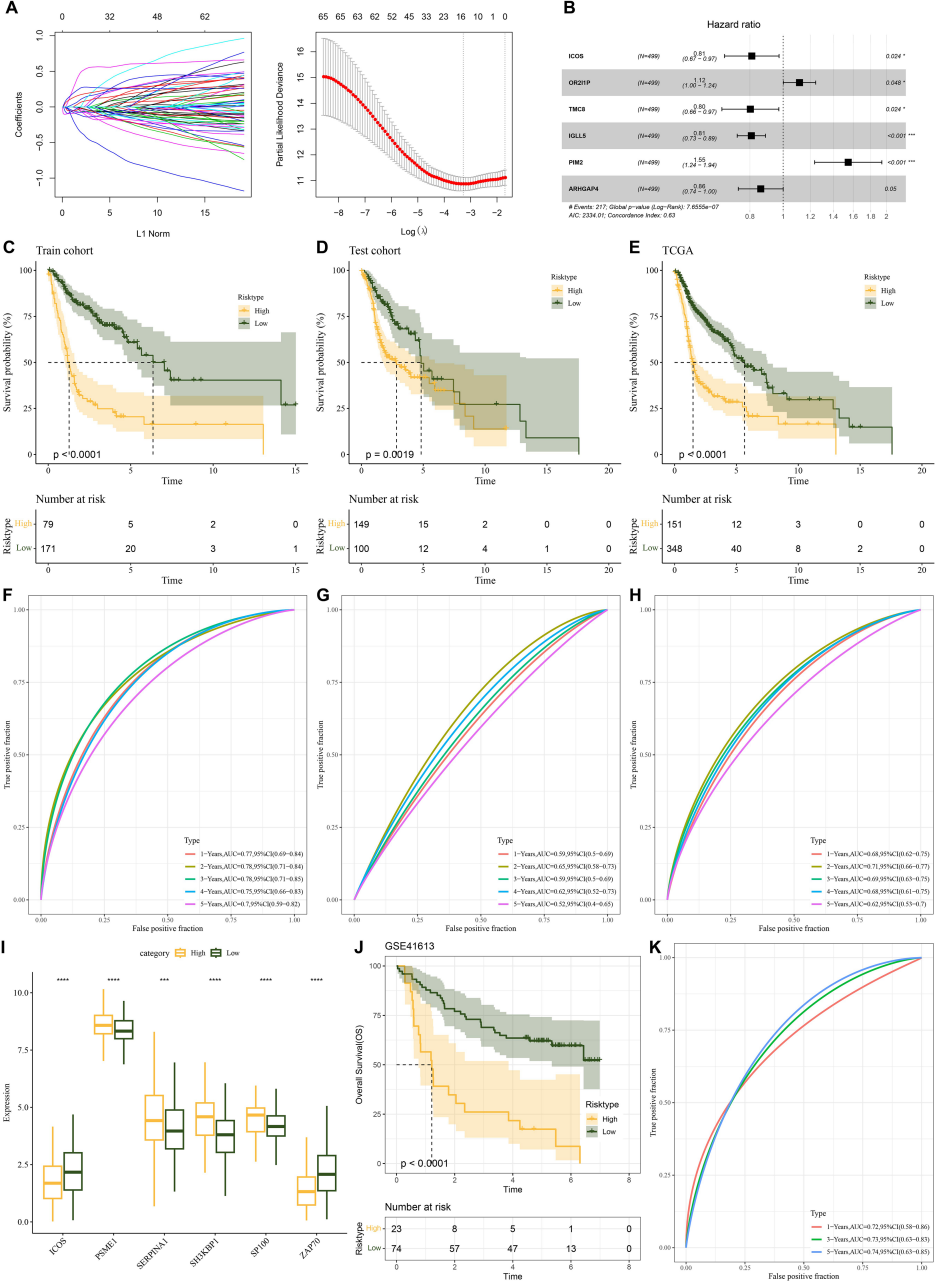
Establishment of a prognosis model for HNSCC patients and validation. **(A)** A number of LASSO Cox shrinkage genes. **(B)** Multifactorial random forest plot. **(C)** KM survival curves was plotted for the TCGA training data cohort. **(D)** KM survival curves was plotted for the TCGA validation cohort. **(E)** KM survival curves was plotted for the TCGA cohort. **(F)** ROC curves for Riskscore in the TCGA training data cohort. **(G)** ROC curve for Riskscore in the TCGA validation data cohort. **(H)** ROC curve of Riskscore in TCGA cohort. **(I)** The expressions of the prognosis genes in the TCGA cohort. **(J)** KM survival curves of Riskscore in the GSE41613 cohort. **(K)** ROC curve of Riskscore in the GSE41613 cohort. ns, *p* > 0.05, not statistically significant; *p < 0.05; ***p < 0.001; ****p < 0.0001.

To create the risk model, we split the TCGA-HNSCC samples into training and test sets in a 5:5 ratio. For LASSO, its effectiveness hinges on the sparsity assumption, which may not hold in the complex genetic landscape of TCGA-HNSC, potentially causing misidentification of prognostic genes and coefficient underestimation (37). Moreover, the data-dependent variable selection process increases the risk of overfitting. As for stepwise regression, the arbitrary selection criteria based on statistical significance, the order-dependence of variable entry/removal, and the susceptibility to overfitting when the gene to sample size ratio is high can all lead to suboptimal model performance and biased results (38). In order to remove redundant confounding genes and identify the genes that have the biggest influence on patients’ prognosis, the R package “survival” was employed to run univariate Cox proportional risk regression on the aforementioned intersecting genes using the training set data. To minimize the gene range in the risk model, we compressed these genes using the “glmnet” package’s LASSO Cox regression approach. To enhance the model generalization, we further performed 10-fold cross-validation (**Figure 3A**). We then used multifactorial stepwise regression analysis to determine which six distinctive genes (*ICOS*, *PSME1*, *SERPINA1*, *SH3KBP1*, *SP100* and *ZAP70*) were independently linked to prognosis (**Figure 3B**). Characteristics indicative of the prognostic outcomes in the TCGA-HNSCC training set were developed based on the expressions of the characterized genes and the regression coefficients as described below: Riskscore = (-0.685*ICOS) +0.749*PSME1 + 0.25*SERPINA1 + 0.41*SH3KBP1 + 0.24*SP100+(-0.368*ZAP70). Based on the best critical value of the Riskscore, TCGA training set patients were classified into low-risk and high-risk groups. KM curves showed that compared with the high-risk group, patients in the TCGA-HNSCC training set (*p*< 0.0001), validation set (*p*< 0.0019), and the low-risk group of the TCGA cohort (*p*< 0.0001) had better overall survival (OS) (**Figures 3C–E**). To investigate the diagnostic accuracy of the prognostic risk model, ROC analyses for 1-, 2-, 3-, 4- and 5-year prognostic predictions were conducted using the “timeROC” R package. The results indicated that the TCGA-HNSCC training set, test set, and TCGA cohort displayed high AUC values at 1-, 2-, 3-, 4- and 5-years (training set: 0.77, 0.78, 0.78, 0.75 and 0.7; test set: 0.59, 0.65, 0.59, 0.62 and 0.52; TCGA cohort: 0.68, 0.71, 0.69, 0.68 and 0.62, **Figures 3F–H**), demonstrating good classification accuracy for prognosis features. Subsequently, we analyzed the expressions of the characterized genes between patients in the low- and high-risk groups of the TCGA cohort. *ICOS* and *ZAP70* were low-expressed in the high-risk group than the low-risk group, whereas *PSME1*, *SERPINA1*, *SH3KBP1* and *SP100* had markedly higher expression in the high-risk group than the low-risk group (**Figure 3I**).

We utilized the GSE41613 dataset to assess the model robustness using comparable models and equivalence coefficients to those utilized in the training set to confirm the stability and dependability of our developed model using NMRG-related signature genes. The training set finding that the prognostic outcomes of high-risk HNSCC patients were more unfavorable (*p*<0.0001, **Figure 3J**) was supported by the validation data. For the 1-, 3- and 5-year periods, the GSE41613 validation set’s AUC values were 0.72, 0.73 and 0.74, respectively **Figure 3K**). For patients with HNSCC, it showed that the prognostic model had good prognostic prediction.

### Mutation characterization in HNSCC high and low-risk groups

**Figure 4:**
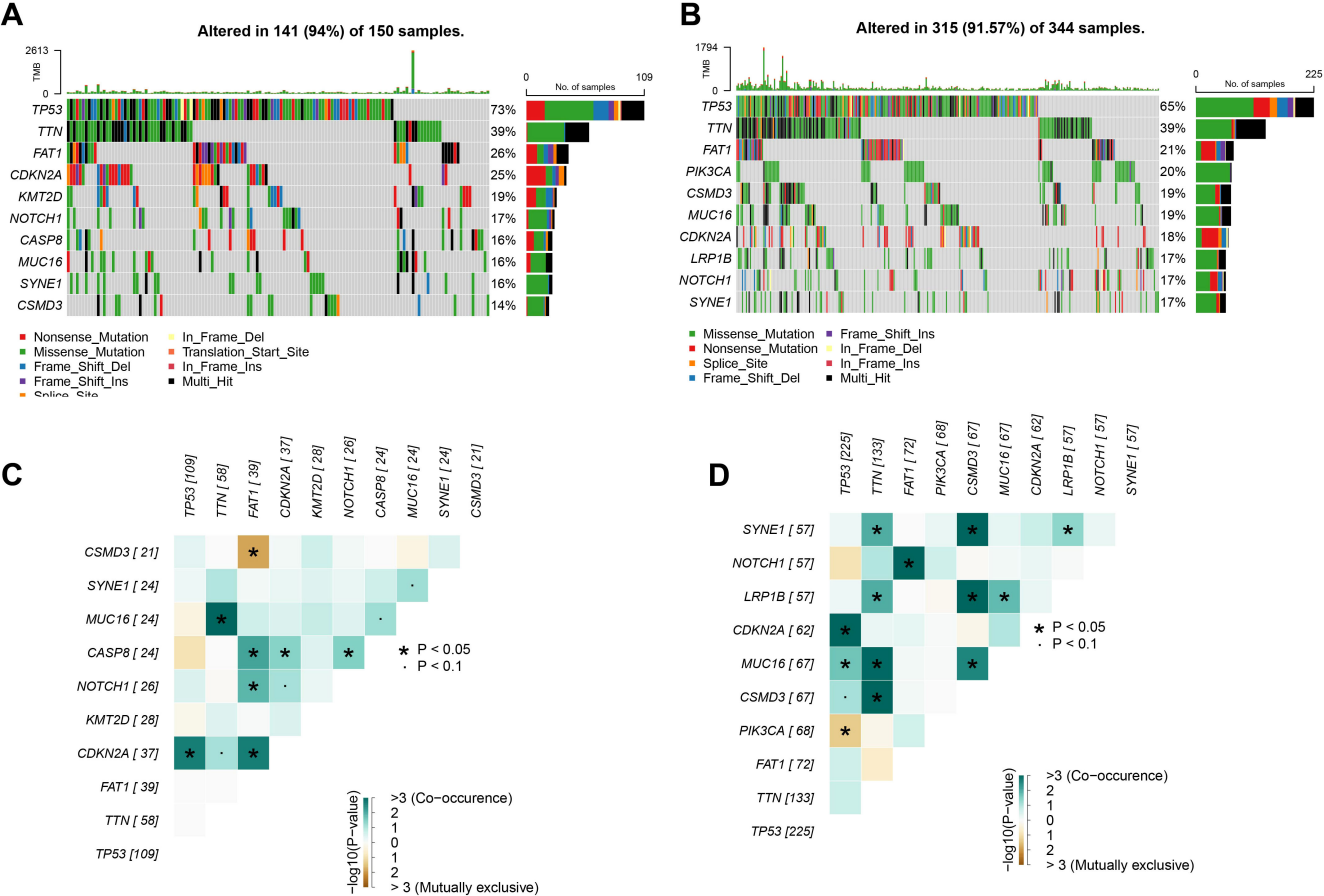
Landscape of gene mutations in HNSCC. **(A)** TCGA cohort high-risk group high frequency mutations top 10 genes. **(B)** TCGA cohort low-risk group high-frequency mutation top 10 genes. **(C)** Interactions of high-frequency mutation top 10 genes in the high-risk group of the TCGA cohort. **(D)** Interactions of high-frequency mutation top 10 genes in the low-risk group of the TCGA cohort. ns, *p* > 0.05, not statistically significant; *p < 0.05.

We further analyzed the gene mutations in the low- and high-risk groups. In the TCGA-HNSCC samples, we observed that in the high-risk group, 141 (94%) HNSCC patients out of 150 samples showed high-frequency mutations top 10 genes, of which the top 3 mutated genes were *TP53* (73%), *TTN* (39%) and *FAT1* (26%) (**Figure 4A**). In contrast, the top 10 mutated genes in HNSCC patients showed mutations in 315 out of 344 samples (91.57%) in the low-risk group, with *TP53* as the gene with the most mutations of 65% in the samples, followed by *TTN* and *FAT1* in 39% and 21% respectively (**Figure 4B**). Subsequently, we revealed the mutational co-occurrence or mutual exclusion patterns between the top 10 mutant gene pairs in different risk subgroups of HNSCC, respectively. Statistical results showed frequent co-occurrence between the *TNN* gene with *MUC16* gene, *CDKN2A* gene with *TP53* gene and *FAT1* genes in the high-risk group (*p* < 0.05, **Figure 4C**). Significant co-occurrence was also shown in the low-risk group, especially between *TTN* and *SYNE1*, *LRP1B*, *MUC16* and *CSMD3* (*p* < 0.05, **Figure 4D**). This may imply that the co-occurring genes have a synergistic role in the associated pathological processes.

### Immunological characterization of HNSCC high and low-risk groups

**Figure 5:**
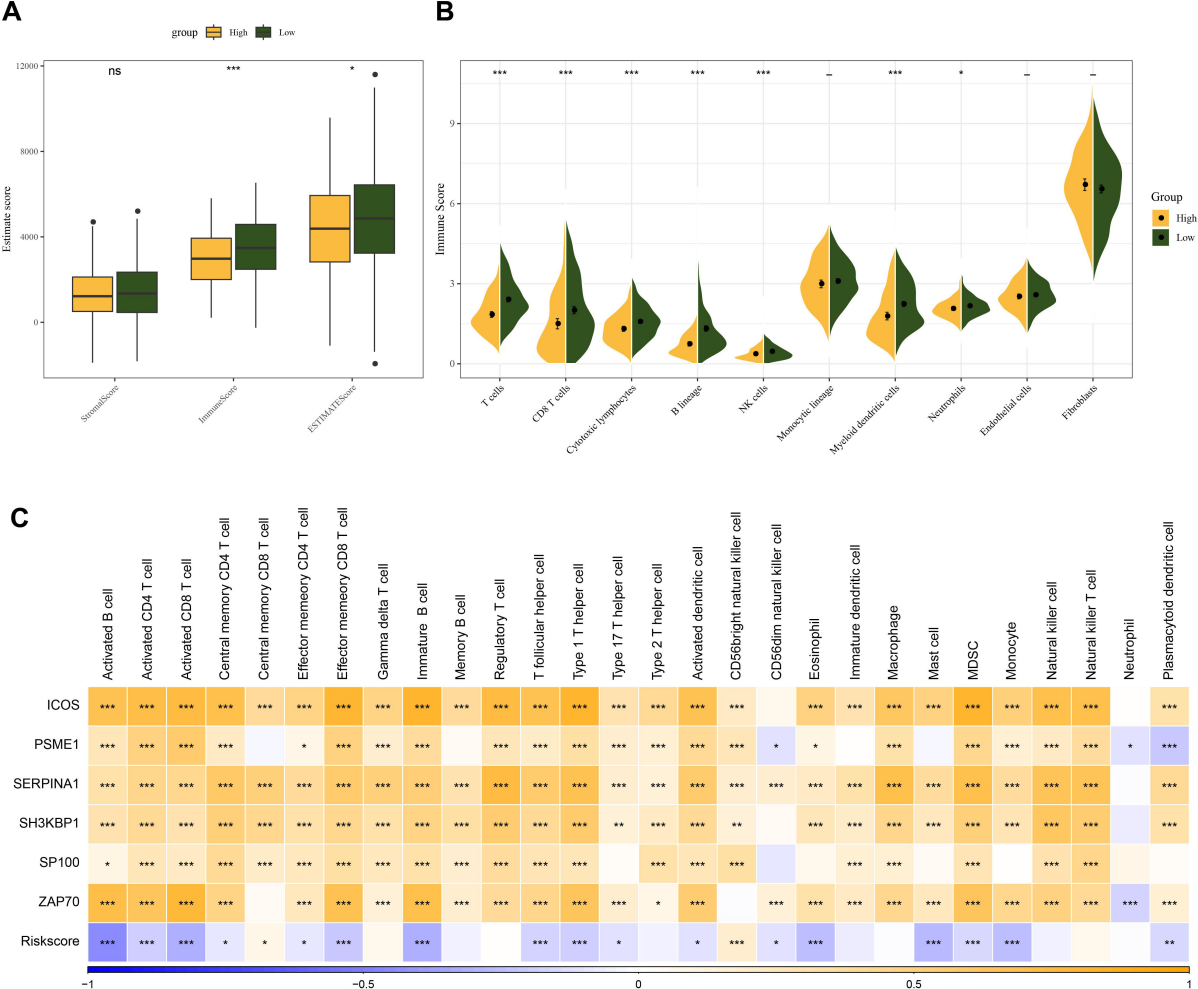
Relationship between Riskscore and HNSCC immune microenvironment. **(A)** ESTIMATE scores between high and low-risk groups in the TCGA cohort. **(B)** MCPcounter assessment of 10 immune cell scores in Riskscore groupings. **(C)** Correlation of immune infiltration scores assessed by ssGSEA with Riskscore and prognostic genes. ns, *p* > 0.05, not statistically significant; *p < 0.05; **p < 0.01; ***p < 0.001.

To analyze the association between the Riskscore and immune microenvironment of HNSCC, we calculated immune cell infiltration using different methods. We first used ESTIMATE algorithm to assess immune cell infiltration and found that the HNSCC high-risk group had lower immune infiltration (**Figure 5A**). Based on MCPcounter, the immune cell score was calculated for the TCGA dataset. The results showed that the myeloid dendritic cells, immune cell scores of T cells, B lineage, CD8 T cells, cytotoxic lymphocytes were all lower in the high-risk group (**Figure 5B**). Using the ssGSEA function of the R package “GSVA”, the scores of 28 types of immune cells in HNSCC were analyzed, and their correlations with Riskscore and signature genes were computed. The data showed that Riskscore was closely negatively linked to the scores of the majority of the immune cells in HNSCC, including MDSC, immature B cells, activated CD8 T cells, mast cells, activated CD4 T cells, activated B cells. On the other hand, the signature genes were positively linked to the scores of the majority of the immune cells in HNSCC, while Riskscore was closely negatively linked to the scores of most of the cells (**Figure 5C**).

### Differences in enriched pathways between high and low-risk HNSCC subgroups

**Figure 6:**
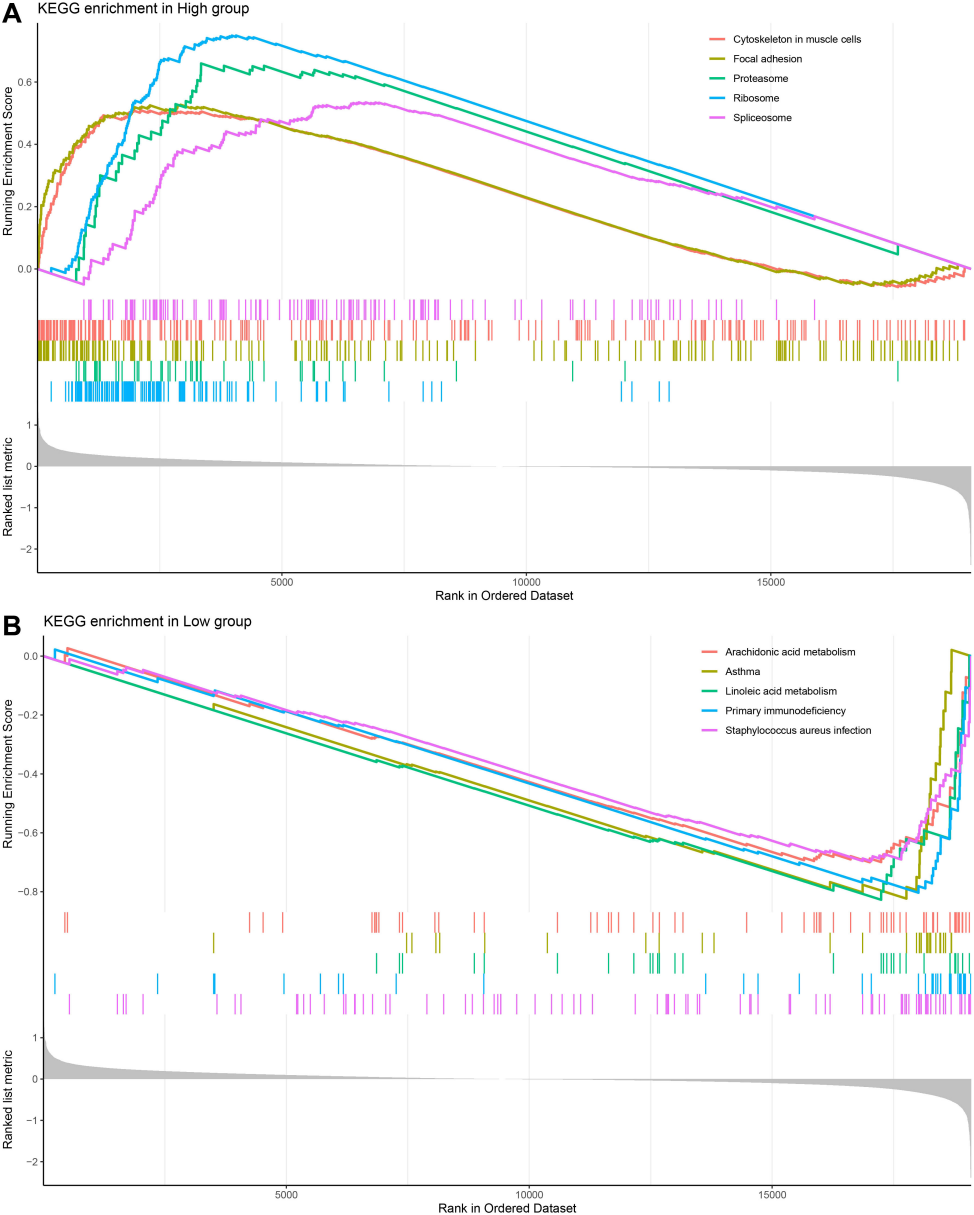
GSEA analysis on differentially expressed genes in different risk subgroups of HNSCC. **(A, B)** KEGG enrichment analysis of HNSCC high and low-risk groups.

To investigate the differences in biological pathways in different risk groups, we performed a KEGG pathway enrichment analysis of DEG between high- and low-risk groups of HNSCC patients. Based on the enrichment results, it was found that the high-risk group was significantly enriched in the pathways of the ribosome, proteasome, and spliceosome (**Figure 6A**). Low-risk group was notably enriched in metabolism-related pathways, including linoleic acid metabolism and arachidonic acid metabolism (**Figure 6B**).

### Downregulation of PSME1 impairs migration and invasion of HNSCC cells

**Figure 7:**
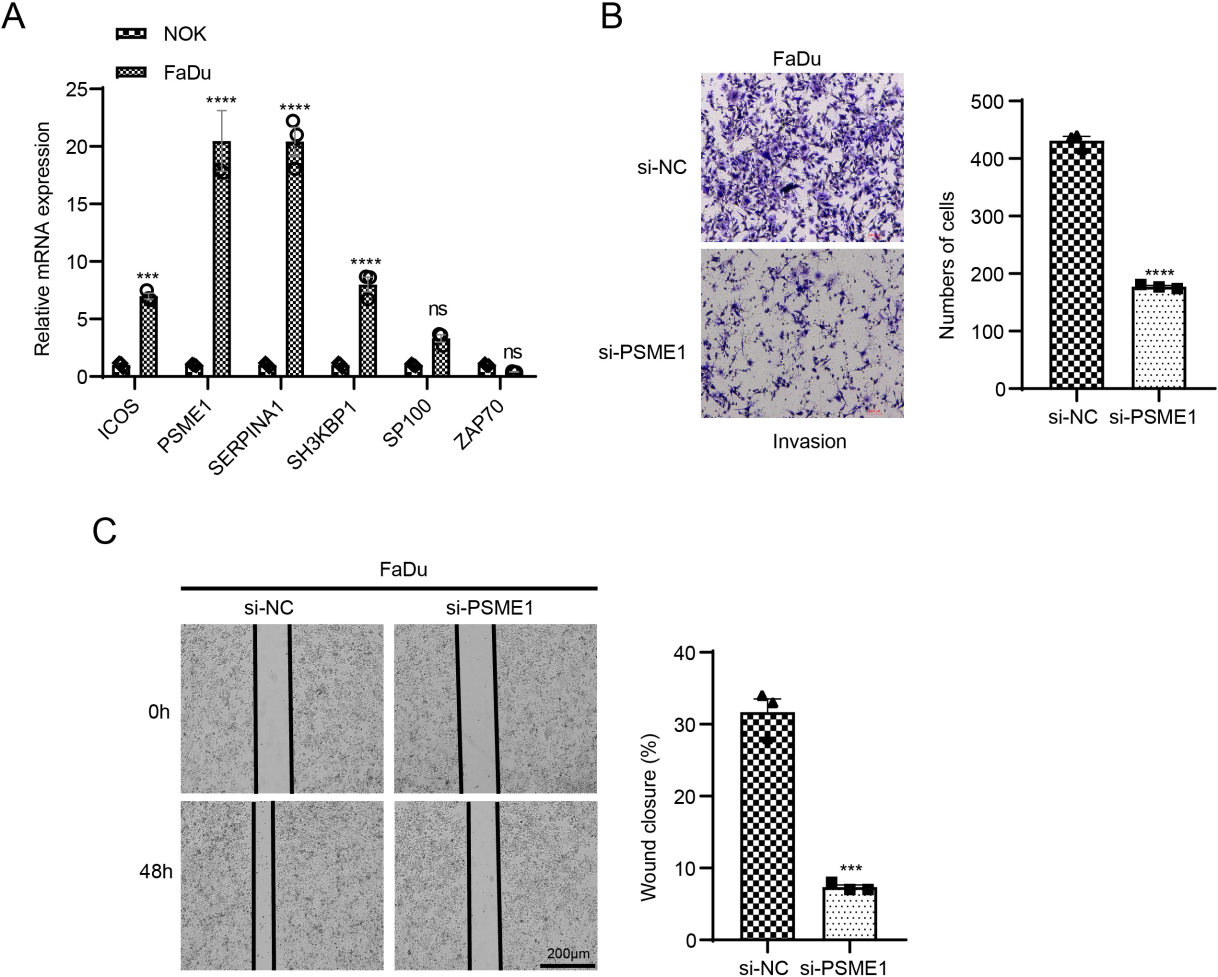
Exploring the biological role of *PSME1* in HNSCC. **(A)** qPCR detection of gene expression levels of *ICOS*, *PSME1*, *SERPINA1*, *SH3KBP1*, *SP100* and *ZAP70* in NOK and FaDu cells. **(B)** Representative images of transwell assay of FaDu cells after *PSME1* knockdown and statistical analysis of invasive cell counts. **(C)** Representative photographs and statistical analysis of wound healing assay in FaDu cells after *PSME1* knockdown. Data are shown as SD ± mean, ns, *p* > 0.05, not statistically significant; ***p < 0.001; ****p < 0.0001.

The mRNA expressions of 6 key genes in NOK and FaDu cells using qPCR. The corresponding results demonstrated that in comparison to NOK cells, the levels of *ICOS*, *PSME1*, *SERPINA1*, and *SH3KBP1* genes were notably upregulated in FaDu cells, while the expressions of *PSME1* and *SERPINA1* genes were higher (**Figure 7A**). It has been reported that *PSME1* has been shown to serve as a therapeutic target in a variety of tumors (39, 40). Therefore, we explored the role of *PSME1* in HNSCC progression. Here, this study analyzed the effect of the *PSME1* gene on FaDu cell invasion and metastasis by wound healing assay and transwell assay. The results showed that the reduction of *PSME1* significantly inhibited the migration and metastasis of FaDu cells (**Figures 7B, C**).

## Discussion

Recent studies have shown that disturbances in NAD^+^ metabolism are associated with cell division, proliferation and apoptosis, all of which can accelerate tumor growth and spread (41). As a result, it is being investigated as a potential tumor treatment. For instance, following examination, scientists discovered that NMRGs accurately evaluated the treatment response and prognostic outcomes for liver cancer patients (42). Furthermore, individuals with stomach cancer who had low levels of NMRG expression often had longer survival times, while those with high levels of NMRG expression had a worse prognosis (43, 44). Therefore, NRMGs also have important research and application value in HNSCC. Here, the present research screened six (*ICOS*, *PSME1*, *SERPINA1*, *SH3KBP1*, *SP100* and *ZAP70*) signature genes independently associated with HNSCC prognosis through integrative analysis. These characterized genes showed good predictability in prognostic assessment in both the training cohort and the validation cohort. They may be mainly involved in defense-related processes such as secretory factor receptor activity and immunity.

These 6 important prognostic genes are strongly linked to carcinogenesis, progression, and treatment, per earlier research in the literature. In particular, tumors expressing high levels of *ICOS* show increased immune cell infiltration, and *ICOS* is a positive prognostic factor in the B7 immune checkpoint co-stimulatory factor family in HNSCC and oral squamous cell carcinoma (45). Advanced-stage cancers had decreased levels of *ICOS* expression, and tumors with high ratios of *PD*-*L1*/*ICOS*, *PD*-*L2*/*ICOS*, or *CD276*/*ICOS* expression have a worse prognosis and a worse prognosis for patients with positive lymph nodes (46). Genes including *PSME1* were found to be positively related to T-cell infiltration and PD-1 expression, in HNSCC, which may influence anti-PD1 treatment efficacy by modulating immune cell infiltration, especially T-cell depletion (39). Potential salivary diagnostic indicators for oral squamous carcinoma include higher salivary levels of *SERPINA1* in individuals with oral squamous cell carcinoma, which correspond with advanced tumor stage (47). Gliomas have high levels of *SH3KBP1*, and these levels have been linked to glioma patients’ lower survival rates. Glioma cell motility, proliferation, and stem cell self-renewal ability are all markedly reduced when *SH3KBP1* is silenced, both *in vitro* and *in vivo* when xenograft tumors are growing (48). Poor clinicopathological characteristics and a poor patient prognosis are directly linked to *SP100* family members with a high expression in pancreatic cancer tissues. Mechanistically, the expressions of *SP100* family members, which are activated in several carcinogenic pathways and strongly co-expressed with M^6^A methylation regulators, are significantly linked with *TP53* mutations (49). It was discovered that HNSCC patients in the low-risk group were more sensitive to immunotherapy in the risk model developed by Liu et al. based on genes like *ICOS* and *ZAP70* (50). *ZAP70* expression was also down-regulated in HNSCC, and its involvement in the predictive risk model of HNSCC patients in the high-risk group revealed a comparatively lower amount of immune cell infiltration and a shorter survival time (51). According to these findings, signature genes may be linked to tumor immune infiltration and treatment, as well as having distinct functions in malignancies. However, based on the analyses that are currently available, they are mostly linked to immune infiltration, invasion and metastasis, cell proliferation, and treatment response, all of which may be useful for predicting prognosis in HNSCC.

*TP53*, *TTN*, and *FAT1* mutation rates were high in both high and low-risk categories of HNSCC patients, according to a gene mutation study. The *KRAS* and tumor protein 53 (*TP53*) genes have been widely exploited as prognostic and predictive gene targets in lung adenocarcinoma because they frequently exhibit notable alterations (52, 53). When cisplatin is used to treat tumors with concurrent *KRAS* and *TP53* mutations, the clinical results are not good (54). Lung cancer is one of the many tumor forms that frequently have mutations in *TTN*, the longest-known gene producing the *TITIN* protein (55). A higher tumor mutational burden and objective reactions to immune checkpoint blockade are closely linked to mutated *TTN*, which is commonly seen in solid tumors (56). In human malignancies, one of the most frequently mutated genes is *FAT1*, which codes for procalcitonin. Loss of *FAT1* function induces a mixed EMT state in human and animal squamous cell carcinomas, which enhances carcinogenesis, progression, invasiveness, stemness, and metastasis (57). The high incidence of somatic TP53 mutations in HNSCC has been linked to tumor progression and decreased survival by preventing cytotoxic CD8^+^ T cell infiltration and encouraging the intra-tumor recruitment of regulatory T cells and M2 macrophages (58). The *TP53*, *FAT1* and *TTN* genes are the most significantly mutated genes in HNSCC, which is in line with our findings. These genes also have high mutation rates in various risk classes of HNSCC patients (59, 60). In conclusion, immune infiltration may be linked to tumor growth mediated by *TP53*, *TTN* and *FAT1* mutations.

The high-risk group of HNSCC has a markedly enhanced ribosome, proteasome, and spliceosome pathway, according to pathway analysis. Pathways like the metabolism of linoleic acid and arachidonic acid were considerably more abundant in the low-risk group. The expression of proteins linked to ribosome biosynthesis was inversely connected with antitumor drug sensitivity and tumor-infiltrating immune cells in HNSCC, and their knockdown prevented cell invasion, migration, and proliferation (61). Protease inhibitors cause the development of proteins with pro- and anti-apoptotic effects because the proteasome controls the expression levels of several proteins with various roles. Signaling proteins and pathways that support cell survival and intrinsic resistance to proteasome inhibitors and other anticancer treatments are typically abnormally activated in cancer cells (62–64). Protease and histone deacetylase co-targeting prevent HNSCC cells from developing acquired treatment resistance (65). HNSCC cell lines exhibit a substantial enrichment of splicing-associated proteins, and splice kinase inhibition dramatically lowers the ability of HNSCC cell lines to invade and form colonies (66). Inhibition of arachidonic acid metabolism results in decreased proliferation and VEGF production in HNSCC cells, whereas its metabolism enhances cancer cell viability (67). Patients with oral cancer have much higher levels of salivary linoleic acid and arachidonic acid, which activate TRPV1 and/or TRPA1 on sensory neurons and contribute to oral cancer pain (68). This implies that tumor immunomodulation, invasion, and metastasis are intimately linked to the variations in pathways between low- and high-risk groups of HNSCC.

CD8^+^ T cells in the tumor microenvironment are well-recognized antitumor immune cells that govern the anticancer response to cytokines and are one of the predictors for indicating immunotherapy success rate for patients (69, 70). Anti-PD-1/PD-L1 therapy by anti-PD-1 antibody or anti-PD-L1 antibody is efficacious by reactivating tumor-infiltrating CD8^+^ T cells in a subgroup of cancer patients. Anti-PD-L1 plus anti-CTLA4 early response in HNSCC is characterized by CD4^+^ T cell activation and recruitment from tumor-draining lymph nodes (71). Anti-PD-1/PD-L1 therapy has been reported to benefit from an increase in tumor-infiltrating cytotoxic T cells (72). Specialized antigen-presenting cells called dendritic cells take samples of the surrounding environment and send co-stimulatory and antigenic signals to adaptive immune system cells (73). In contrast, mast cells are innate immune cells found in human tissues that control tumor cell growth and angiogenesis to modify the inflammatory response and TME homeostasis in patients (74). According to reports, cancer immunotherapy can help stimulate tumor-specific cytotoxic T cells inside the lymphoid organs, increase the activity of cytotoxic T lymphocytes within the tumor, and create potent and durable anti-tumor immunity. To maximize the quantity and caliber of the cytotoxic T lymphocyte response, some dendritic cells will assist in the signaling process from CD4^+^ T cells to CD8^+^ T cells during initiation (75). Analysis of the immune microenvironment based on the predictive model of this study showed that the abundance of CD8 T cells, mast cells, T cells, B lineage, cytotoxic lymphocytes, and myeloid dendritic cells infiltration was markedly upregulated in the low-risk group. The upregulation of the level of infiltration of these immune cells in low-risk patients predicted a higher level of immunity in the tumor microenvironment, which is an advantage of immunotherapy.

NAD^+^ levels can be modulated through dietary intake of NAD^+^ precursors, such as nicotinamide (NAM), by inhibiting enzymes that consume NAD^+^, and by regulating the activity of enzymes involved in NAD^+^ biosynthesis (76). Clinical trials demonstrate that the oral administration of NAM exhibits chemopreventive properties in the development and recurrence of skin squamous cell carcinoma among both high-risk immunocompetent individuals and those with compromised immune systems (77, 78). Furthermore, NAM has been shown to enhance the effectiveness of radiotherapy for HNSCC and laryngeal squamous cell carcinoma (79, 80). Current trials are investigating the efficacy of NAM when used in conjunction with targeted therapies in patients with advanced non-small cell lung cancer (81). Further a prognostic NAD^+^ metabolism-related gene signature has been developed to predict the response to immune checkpoint inhibitor in glioma (82).

However, there are still certain limitations in this study. First, this study relies heavily on publicly available databases for its analysis, and although representative, the sample size is relatively limited and sample heterogeneity exists. Therefore, we will incorporate multi-center, large sample size clinical cohorts in future studies, combined with prospective follow-up data, to improve the robustness and generalization ability of the model. In addition, although a prognostic model based on NMRGs was constructed and the cellular function of *PSME1* was experimentally validated, there is a lack of mechanistic studies on the functions of other characterized genes. In the future, the functional roles of the characterized genes in HNSCC can be verified by *in vivo* experiments (including knockout/overexpression models, signaling pathway analysis, etc.) and their regulatory mechanisms can be revealed. Finally, immune infiltration analysis is mainly based on algorithmic estimation at the transcriptome level. Follow-up studies may combine tumor tissue samples for immunostaining, mass spectrometry flow cytometry, and other techniques to further confirm the immune cell infiltration status and its true correlation with characteristic genes.

Future research may explore several avenues. Firstly, it is essential to conduct thorough investigations into the mechanisms of the six identified genes. Understanding how these genes influence tumor development through both *in vitro* and *in vivo* experiments will elucidate their specific roles in HNSCC. Secondly, given the relationship between gene expression and immune cell infiltration, it is important to examine the dynamic interactions involved in disease progression within the tumor microenvironment. Longitudinal studies that monitor changes in gene expression related to immunity could uncover new therapeutic targets. Lastly, integrating multi-omics data can enhance our understanding of the heterogeneity of HNSCC and assist in identifying more precise biomarkers.

From a clinical perspective, our findings hold significant implications. The developed risk model can categorize patients effectively, enabling personalized treatment approaches: high-risk patients may benefit from aggressive treatment options, while low-risk patients can avoid unnecessary side effects. Additionally, genes associated with immune cell infiltration have the potential to predict responses to immunotherapy, assisting patients in selecting appropriate treatment options. Moreover, developing and testing pathway-specific drugs that target pathways prevalent in various risk groups could offer new avenues for treatment in clinical trials.

## Conclusion

This study used machine learning methods and identified six feature genes independently linked to HNSCC prognosis and established a prognosis model. The robustness of the model was confirmed, and it can stably assess the prognosis and immune infiltration of HNSCC patients, contributing to the personalized treatment of the cancer. This study could offer a reference for further investigation into the potential biomarkers for diagnosis and prognosis prediction in HNSCC patients.

## Data Availability

The original contributions presented in the study are included in the article/supplementary material. Further inquiries can be directed to the corresponding author.
